# Reducing IP Turnover: What Leaders Can Do to Keep Their Infection Preventionists

**DOI:** 10.1017/ash.2025.225

**Published:** 2025-09-24

**Authors:** Becca Bartles, Sara Reese

**Affiliations:** 1 APIC

## Abstract

**Background:** Turnover within the field of Infection Prevention is high, and this can cause significant organizational disruption and associated costs. Career growth and professional development are important retention factors in the field of infection prevention, however many infection preventionists (IP) tend to find their roles become stagnant resulting in attrition. The objective of this study was to determine organizational and leadership factors that contribute to IPs staying with an organization while still growing their career and profession. **Methods:** A mixed-methods approach with focus groups and survey methodology was used to assess organizational and leadership factors. Focus group and survey participants were stratified by (1) IPs who had been with the same organization for >5 years (long-term IPs), (2) IPs who have changed from one IP role to another IP role within the last 3 years (short-term IPs) and (3) IP leaders. Survey responses were analyzed using a Cochran Mantel-Haenszel test for associations. Qualitative responses were analyzed using a thematic analysis to identify themes and subthemes. **Results:** There were 82 participants in the focus groups and 632 survey respondents. 37.5% (n=117) of long-term IPs responded that their organizations were effective at providing career advancement opportunities in infection prevention compared to only 16.1% (n=20) of short-term IPs (p) **Conclusion:** The findings suggest that healthcare organizations could reduce turnover amongst IPs by providing career advancement opportunities and supporting professional growth. Future studies should focus on identifying the most effective professional advancement pathways specific to the field of infection prevention and control.

